# Editorial: Youth, Young People, and Sport in the Twentieth Century

**DOI:** 10.3389/fspor.2022.887919

**Published:** 2022-04-25

**Authors:** Patrick Clastres, François Vallotton, Nicolas Bancel

**Affiliations:** University of Lausanne, Lausanne, Switzerland

**Keywords:** sport history, youth-young adults, twentieth century, Western Countries and non, masculinization, sport policies, students, scouting

The ten contributions in this volume were presented at the 23rd Congress of the European Committee for Sports History (CESH) organized in September 2019 by the University of Lausanne's Institute of Sport Sciences (ISSUL) and the Social and Political Sciences Faculty (SSP). Our topic was chosen in response to the fact that, even though sport is commonly associated with youth, few sports historians have focused on the issue of age. Indeed, research into the history of sport has tended to concentrate on aspects such as sportspeople's/spectators' national, social, gender or racial identities, rather than on age/age groups. There are, of course, exceptions to this rule in fields such as the history of physical education, the history of bodily and moral discipline policies and the history of supporters. Historians, sociologists, ethnologists and anthropologists have shown that the ages at which childhood and youth begin and end vary from one civilization to another, from one social category to another, from one gender to another, and from one era to another.

According to Mauger (2001), by declaring in 1978 that “youth is only a word,” Bourdieu (1978) was encouraging us to analyse the social and cultural differences between youths and not to see young people as “a social unit, a constituted group, with common interests, related to a biologically defined age,” but as “an age of life”, i.e., a sequence of biographical trajectories. Our preliminary questioning owes much to the work published in 1996 by Levi and Schmitt (1996) who affirmed that “the word “youth” has meaning everywhere and always, even if it takes on different names and contents depending on the period” and “that among the common characteristics, we can retain the transitory status, intermediate between childhood and adulthood, a brief period of learning (sexual, warlike, professional, etc.) and finally a status of *margin*.”

So our general question was: What is the case from a sports perspective? And the crucial questions that arise is first of all that of sources. As Sirinelli (2009) writes for the periods prior to the nineteenth century, “for a long time a sociologically dominated category, youth was most often barely audible to contemporaries and the traces left about it were external, coming, for example, from public authorities or from the sphere of knowledge development.” In the light of documents as rich as the *Carnets de voyage dans les îles Britanniques* (Clastres, 2023) or the *Mémoires de jeunesse de Pierre de Coubertin* (Clastres, 2008), or even the Diary of the 1896 Olympic tennis champion John Pius Boland (Gillmeister, 2008), it would be interesting and useful to systematically collect the testimonies left by the young practitioners themselves.

At what age do children start and stop doing sport in different societies? Does giving up sport signal entry to the adult world? Or into the world of seniors, considered too old for physical exercise? Could doing sport be seen as a heuristic criterion for defining the transitional state of youth? Does one stay young into old age as long as one continues doing sport? Do boys leave the often-maternal world of childhood games by adopting masculine sports rituals? Do girls adopt new forms of exercise as they get older? Does physical exercise play a role in the strict separation between girls and boys or is there a degree of porosity? Where there are age distinctions with respect to sports, how did they arise? Do these age categories reflect age categories in the rest of society (puberty, school, work, religion, citizenship), or are they specific to the world of sport? How and by whom are they defined and justified?

Is it possible to identify “youth sports groups,” that is, communities of young people whose identity is based on their young age and their sports activities? What becomes of these communities of young sportspeople when their members become adults? Do they continue, dilute, evolve, break up, reactivate? Do young sportspeople feel particular emotions that are specific to their age? Once they become adults, how much nostalgia do they retain for their sporting pasts? Do young sportspeople see themselves primarily as sportspeople or as young people? Is there a methodological risk in reducing them to a single category, that of “sportsperson,” when they also play other social roles in their daily lives? How, here like elsewhere, can age, social position and gender be articulated? What part do young people play in the institutions that run sport? Do young people take the initiative to create clubs, leagues or federations? In the case of institutions created and controlled by adults, are young people's voices heard. How are they viewed?

In terms of other forms of power exercised by adults over sporting practices (political, medical, educational, military, religious, professional), what impact do they have on young people? How much freedom do they truly have? Is it, in fact, possible to explain the success of sport across the centuries by the freedom young people draw from the statute of being a sportsperson? But, paradoxically, do not these practices also, and sometimes simultaneously, have a normative, and/or disciplinary dimension?

## Young People and Sport in Non-Western Countries

In this volume, we also focus on a number of studies dealing with the various sports activities intended for young people in non-Western regions and we wish to recall here the double contribution of British, American and French historiographies.

Since the mid-1980s, Mangan (1986, 1989) has taken an interest in the issue of sport within the British empire, in which young people are the main actors. Research on the articulation between sport and religion has been carried out on a large scale, going beyond the role of Protestantism only, as exemplified by the work of Alpert and Remillar (2019). Inaugurating the tradition of the Postcolonial Studies in sports history, Appadurai (1996) highlighted in the development of cricket in colonial India. Anglo-Saxon historians did also investigate the Pacific region like Sacks (2019). American researchers claim a global vision of the mechanisms underlying the dissemination of sport among the young in colonized countries, like the synthesis on Games and empires directed by (Guttmann, 1994). Both Guttmann (1994) and Appadurai (1996)—while integrating the writings of globalist Galtung (1991) evaluating “the sport system as a metaphor for the world system”—point out the hybridization of sports practices by young athletes during the diffusion of cricket throughout India. The valorization of the American research benefits from the efficient relay constituted by the *Journal of Sport History*. In volume 33, nr 1, published in 2006, this journal applies a comparative perspective, entitled “Globalization as imperialism?,” to three case studies focused on American, German (Pfister, 2006) and French (Combeau-Mari, 2006) colonization. Although Anglo-Saxon sports were received with a certain amount of reserve in China, they achieved a break-through owing to the militancy of the YMCA (Gems, 2004). Researchers such as Cleveland et al. (2020) are currently pursuing these perspectives with regard to the evolution of sport in Africa.

Considering the francophone historiography, we must consider two pioneering works: Youssef Fatès's new light on the cultural history of Algeria's decolonization (1994; 2002; 2020), building on the pioneering work of Kaddache (1976, 2003), and Bernadette Deville-Danthu's (1997) PhD thesis defended two years before on the diffusion of military and school gymnastics and modern sports in French Black Africa. This research was further developed by Bancel (1999) in his thesis on youth movements and modern sports in the same area. As for the African Great Lakes region, we should mention Thomas Riot's thesis on colonial Riot (2011). In their IJHS special issue on “post-colonial sports,” Frenkiel et al. (2015) continued this research along the same lines. Several other studies present a similar comparative approach. A relevant example is the special edition of *Outre-mers. Revue d'histoire* entitled “Le sport dans l'empire français, un instrument de domination coloniale ?” under the direction of (Abassi, 2009). Addressing the issue of “cultural globalization” initiated by Singarevelou and Sorez (2010) revisited the question of the diffusion and local adaptation of modern sports by their young practitioners.

## From Scouting in Ivory Coast to Soviet Young Elite Athletes

This collection of 10 articles covers a wide chronological spectrum, from the second half of the nineteenth century to the present day, and various European and non-European national realities.

The role of sport in the acculturation process of youth is first illustrated by the example of physical education in Prague, which highlights its essential role in both healthcare and military terms (Waie and Pavlu). Beyond the strict domain of primary education, the Swiss example shows the role of gymnastics clubs in the extension of the phenomenon both in terms of geographical and political areas and age groups (Mayencourt and Quin). With the turn of the nineteenth and twentieth centuries, the question of socialization through sport is approached via cultural mediators whose youth is an asset (Bourmaud) but also, in the case of Theodore Roosevelt and his son hunting in Africa, as the stake in a process of learning cultural as well as moral values (Chasles). The crossroads between sport, youth and war are dealt through the voluntary commitment of a young Spanish swimmer, member of a workers' club, who joins the Republican camp at the time of the Civil War (Viuda-Serrano and Ibarrondo-Merino).

Two contributions address the propagandist role of sport through the examples of the High School Secondary Students created by the Peron government in 1953 (Hémeury) and the scouting movement in Côte d'Ivoire before and after independence (Nicolas). However, their impact is balanced in the first case by the autonomy of the institution from political struggles, and the resistance that can be observed within the scout association in the second. The Cold War issue is analyzed also in two articles. The first one highlights the role of sport within the World Festivals of Youth and Students which embodies a form of cultural diplomacy of the USSR and the communist bloc (Lesnykh). The second one focuses on the representations of Soviet elite sport in its consequences for young athletes: beyond the Western narrative on the making of champions, the contribution underlines the need for a broader contextualization of these issues (Dufraisse). Finally, a contemporary ethnographic analysis of the practice of twirling in Switzerland, a practice with little legitimacy that reinforces female stereotypes, underlines the need to take into account the gendered dimension in order to approach the general theme (Manh Ly).

## Author Contributions

PC organized the CESH2019 Congress in Lausanne and designed this special issue. NB wrote the first part of the Editorial. PC and FV edited most of the articles. NB wrote the paragraph titled “Young people and sport in non-western countries” and FV the one called “From scouting in Ivory Coast to Soviet Young Elite Athletes”. All authors contributed to the article and approved the submitted version.

## Conflict of Interest

The authors declare that the research was conducted in the absence of any commercial or financial relationships that could be construed as a potential conflict of interest.

## Publisher's Note

All claims expressed in this article are solely those of the authors and do not necessarily represent those of their affiliated organizations, or those of the publisher, the editors and the reviewers. Any product that may be evaluated in this article, or claim that may be made by its manufacturer, is not guaranteed or endorsed by the publisher.
